# An updated meta-analysis of the anti-methanogenic effects of monensin in beef cattle

**DOI:** 10.1093/tas/txae032

**Published:** 2024-03-09

**Authors:** Reinaldo F Cooke, Lidiane R Eloy, Sheila C Bosco, Pedro V F Lasmar, José M C de Simas, Tiago Leiva, Sergio R de Medeiros

**Affiliations:** Department of Animal Science - Texas A&M University, College Station, TX 77845, USA; Analisa Soluções em Estatística, Porto Alegre, RS 90010-000, Brazil; Analisa Soluções em Estatística, Porto Alegre, RS 90010-000, Brazil; Elanco Animal Health, São Paulo, SP 04703-002, Brazil; Elanco Animal Health, São Paulo, SP 04703-002, Brazil; Elanco Animal Health, São Paulo, SP 04703-002, Brazil; Embrapa Pecuária Sudeste, São Carlos, SP 13560-970, Brazil

**Keywords:** beef cattle, meta-analysis, methane, monensin

## Abstract

Meta-analyses were performed to quantitatively summarize the effects of monensin on in vivo methane (CH4) production in beef cattle, and differentiate these outcomes according to dietary management, dose of monensin, and length of monensin supplementation. Data from 11 manuscripts describing 20 individual studies were used, and CH4 was converted to g/d when required. Studies were classified according to dose of monensin (mg/kg of diet dry matter), length of monensin supplementation prior to the last CH4 measurement, feeding management (ad libitum vs. limited-fed), and diet profile (high-forage or high-concentrate diets). Variance among studies were assessed using a χ² test of heterogeneity and calculated using *I²* statistics. The inclusion of monensin decreased (*P *< 0.01) CH4 production by 17.5 g/d when all studies were analyzed together. A moderate (*P *< 0.01) heterogeneity (*I²* = 55%) was detected for CH4 production estimates between studies; thus, meta-analyses were performed within classes. The reduction in CH4 differed (*P *< 0.01) according to dose of monensin, as it decreased (*P *< 0.01) by 25.6 g/d when the high recommended dose range was used (32 to 44 mg/kg), and tended to decrease (*P *≤ 0.07) by 9.7 and 13.5 g/d when the moderate (≤31 mg/kg) and above recommended (≥45 mg/kg) doses were used, respectively. The reduction in CH4 also differed (*P *< 0.01) according to the length of monensin supplementation. Monensin decreased (*P *≤ 0.05) CH4 production by 24.3 g/d when supplemented for <15 d, by 15.4 g/d when supplemented from 23 to 33 d, by 24.3 g/d when supplemented from 52 to 79 d, and tended to decrease (*P *= 0.06) CH4 production by 3.21 g/d when supplemented from 94 to 161 d. The reduction in CH4 did not differ (*P *= 0.37) according to diet profile, despite a 30% difference in reduction when monensin was added to high-forage (20.89 g/d) compared with high-concentrate diets (14.6 g/d). The reduction in CH4 tended to differ according to feeding management (*P *= 0.08), decreasing by 22.9 g/d (*P *< 0.01) when monensin was added to diets offered ad libitum, and by 11.5 g/d (*P *= 0.05) in limit-fed diets. Collectively, this study provides novel insights and further corroborates monensin as CH4 mitigation strategy in beef cattle operations. The most effective responses were observed during the first 79 d of monensin supplementation, and when monensin was included between 32 to 44 mg/kg of diet, was added to high-forage diets, and added to diets fed ad libitum.

## Teaser

Climate change is a major challenge to the environment and humanity, and reducing methane emissions is critical to mitigate global warming. This study provides novel insights into monensin as a nutritional strategy to reduce methanogenesis in beef cattle.

## Introduction

Climate change is a major challenge to the environment and humanity, and reducing the emission of greenhouse gases (GHG) is critical to mitigate global warming (Kweku et al., 2018). Methane (CH4) is a GHG with warming potential 25 × greater than CO_2_ and livestock production contributes to 30.7% of the overall anthropogenic CH4 emissions (UNEP CCAC, 2021). Enteric CH4 production is primarily regulated by ruminal carbohydrate fermentation and subsequent volatile fatty acid synthesis, which impacts the availability of hydrogen for CH4 formation (Ellis et al., 2008). Therefore, management interventions that modulate these mechanisms can be used to alleviate CH4 emissions from beef production systems.

Monensin is a carboxylic polyester ionophore widely used to enhance feed efficiency in beef and dairy cattle (Marques and Cooke, 2021). Ionophores inhibit gram-positive bacteria and favor propionate production, while mitigating ruminal proteolysis and ammonia synthesis (Goodrich et al., 1984; Duffield et al., 2012). Monensin has also been shown to reduce ruminal methanogenesis by limiting the availability of hydrogen and formate, which are primary substrates for methanogenic bacteria (Bergman, 1990; Ellis et al., 2012). For these reasons, monensin supplementation has been considered as a CH4 mitigation strategy in beef cattle operations (Beauchemin et al., 2008).

Several studies investigated the anti-methanogenic effects of monensin on cattle, and results were used in a meta-analysis by Appuhamy et al. (2013). These authors reported that monensin reduced CH4 emissions in beef cattle by 19 g/d based on results from 11 published studies. Appuhamy et al. (2013) also suggested that dose of supplementation, length of supplementation, and dietary composition can influence the anti-methanogenic effects of monensin. Persistency of CH4 mitigation is critical for validation of nutritional interventions (Van Zijderveld et al., 2011), whereas dietary management (i.e., ad libitum vs. limited-fed, forage-based vs. grain-based diets) influence monensin dosage and its CH4 mitigation properties (Guan et al., 2006; Beauchemin et al., 2008). More recent analyses on the anti-methanogenic effects of ionophores are still warranted (Kelly and Kebreab, 2023), as Appuhamy et al. (2013) evaluated beef cattle studies from 1981 to 2006. Therefore, we hypothesized that an updated meta-analysis would provide novel insights into the anti-methanogenic potential of monensin to beef cattle. This study quantitatively summarized the effects of monensin on in vivo CH4 production in beef cattle, and differentiated these outcomes according to dietary management, dose of monensin, and length of monensin supplementation.

## Materials and Methods

This meta-analysis was conducted using the PICOT methodology (Richardson et al., 1995), and the population under consideration was beef cattle. The intervention and comparator of interest were beef cattle supplemented with monensin vs. beef cattle not supplemented with monensin (control). The primary outcome of the review was in vivo CH4 production.

### Literature Search and Data Extraction

A systematic database was constructed by conducting a comprehensive search of the literature via PubMed, Science Direct, Scopus, Web of Science, CAB Direct, Scielo, and Google Scholar. The systematic search considered the terms (1) beef cattle, (2) methane, (3) CH4, and (4) monensin. The database ranged from 1970 to 2023, and articles in English, Portuguese, and Spanish were considered. Arrangement and extraction of data were performed according to the PRISMA procedure (Moher et al., 2009). The search yielded 11,765 articles, whereas six additional articles were identified through bibliographic review. The database was reduced to 9,022 articles following initial screening and removal of duplicates. Articles were then individually evaluated by title and abstract, resulting in removal of 8,929 articles. The remaining 93 articles were further screened for eligibility, and 82 were removed for reasons such as not evaluating beef cattle (*n* = 20), using in vitro techniques (*n* = 25), not evaluating CH4 (*n* = 11), and for not evaluating monensin (*n* = 6). The final database contained 11 manuscripts describing 20 individual studies (including 11 individual studies used by Appuhamy et al. [2013]), which were used for subsequent analysis (Table 1). McGinn et al. (2004) and Mwenya et al. (2004) evaluated Holstein steers receiving growing diets relevant to beef cattle production, and for this reason, were used herein and by Appuhamy et al. (2013). All studies used Rumensin^®^ (Elanco Animal Health, Greenfield, IN) as the source of monensin.

**Table 1.** Sources and characteristics of studies used in the meta-analyses

Reference	Dose^1^	Length^2^	Diet profile^3^	Diet management
Thornton and Owen (1981) to study 1	41	15	50:50	Limit-fed
Thornton and Owen (1981) to study 2	54	15	20:80	Limit-fed
Thornton and Owen (1981) to study 3	41	15	67:33	Limit-fed
Wedegaertner and Johnson (1983) to study 1	32	33	20:80	Ad libitum
Wedegaertner and Johnson (1983) to study 2	53	33	20:80	Limit-fed
Rumpler et al. (1986) to study 1	55	23	20:80	Limit-fed
Rumpler et al. (1986) to study 2	55	23	20:80	Limit-fed
Rumpler et al. (1986) to study 3	55	23	20:80	Limit-fed
O’Kelly and Spiers (1992) to study 1	33	52	100:0	Ad libitum
O’Kelly and Spiers (1992) to study 2	33	52	100:0	Limit-fed
McGinn et al. (2004)	33	21	75:25	Ad libitum
Mwenya et al. (2004)	24	22	20:80	Ad libitum
Guan et al. (2006) to study 1	33	70	86:14	Ad libitum
Guan et al. (2006) to study 2	33	70	31:69	Ad libitum
Neto et al. (2009)	38	25	100:0	Ad libitum
Tomkins et al. (2015) to study 1	10	26	100:0	Ad libitum
Tomkins et al. (2015) to study 2	53	26	100:0	Ad libitum
Hemphill et al. (2018)	19	161	90:10	Limit-fed
Vyas et al. (2018) to study 1	33	79	65:35	Ad libitum
Vyas et al. (2018) to study 2	33	94	8:92	Ad libitum

^1^Dose of monensin used as mg/kg of diet dry matter.

^2^Days receiving treatments prior to methane measurement.

^3^Forage to concentrate ratio (dry matter basis).

Mean CH4 production in control vs. monensin-treated cattle was the main variable of interest, and was extracted as mean ± standard deviation. Production of CH4 was converted to g/d when required, assuming that a mole of CH4 weighs 16 g and has a volume of 22.4 L (Appuhamy et al., 2013). Dose of monensin (mg/kg of diet DM), days receiving treatments prior to CH4 measurement, feeding management (ad libitum vs. limited-fed), and diet profile (high-forage or high-concentrate diets) were considered based on our hypothesis. To facilitate analyses, dose of monensin, days receiving treatments, and diet profile were converted into classes. Doses were classified according to the recommended levels for improving feed efficiency in beef cattle (5.5 mg/kg to 44 mg/kg of the diet, dry matter basis [DM]; Duffield et al., 2012) as moderate recommended dose (MOD; <31 mg/kg), high recommended dose (HI; 32 to 44 mg/kg), and above recommendation (ABV; above 44 mg/kg). Diets were classified as high-forage (<50% concentrate inclusion, DM basis) or high-concentrate diets (≥50% concentrate inclusion, DM basis). Days receiving monensin until CH4 evaluation were classified as short length (15 d), moderate length (23 to 33 d), long length (52 to 79 d), and extended length (94 and 161 d) according to ruminal dynamics in adapting to dietary changes (14 d; Brown et al., 2006). Only the last measurement in studies that assessed CH4 over multiple times was used to evaluate persistency of CH4 mitigation (Appuhamy et al., 2013).

### Statistical Analysis

The presence of publication bias was assessed using the enhanced contour funnel plot and standard error as measurement of study size (Sterne and Harbord, 2004). The Egger’s regression test for funnel plot asymmetry (Egger et al., 1997) was performed using the *metabias* function of the meta package in R (version 1.4.1717; R. Foundation for Statistical Computing, Vienna Austria). Meta-analyses were performed using the *metacont* function of the meta package in R (version 1.4.1717; R. Foundation for Statistical Computing).

The effect size was calculated using mean difference (MD; mean of monensin treatment—mean of control treatment). The effect size of each study was adjusted (weighed) according to the inverse of its variance (Lean et al., 2009). Variance among studies were assessed using a χ² test of heterogeneity (Egger and Smith, 2001) and heterogeneity was calculated using *I²* statistics, which quantifies the impact of heterogeneity in a meta-analysis using mathematical criteria that are independent of the number of studies and treatment effect metrics (Higgins and Thompson, 2002). The *I²* is a transformation of the square root of χ² divided by its degrees of freedom, and reflects the proportion of the total variation in each study caused by heterogeneity (Higgins et al., 2003). Negative *I²* are assigned a value of 0% and the following criteria were adopted according to I² value (Higgins et al., 2003): 0% = no heterogeneity between studies, 25% = low heterogeneity, 50% = moderate heterogeneity, 70% to 100% = substantial heterogeneity (Rabiee et al., 2010). The τ² statistics was also generated as an estimate of the variance between studies in a meta-analysis of random effects (Higgins and Green, 2011), using the Hartung–Knapp adjustment method to evaluate the statistical significance of the pooled effect estimate (Hartung and Knapp, 2001).

## Results and Discussion

**Figure 1.**
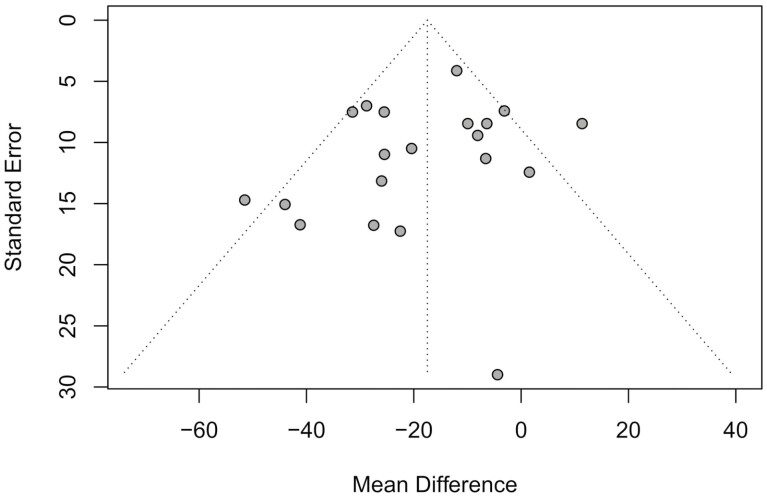
Funnel plot for the effects of monensin supplementation on in vivo methane production (g/d) in beef cattle. The Egger’s regression test for funnel plot asymmetry was not significant (*P *= 0.25), indicating no publication bias (Egger et al., 1997).

**Figure 2.**
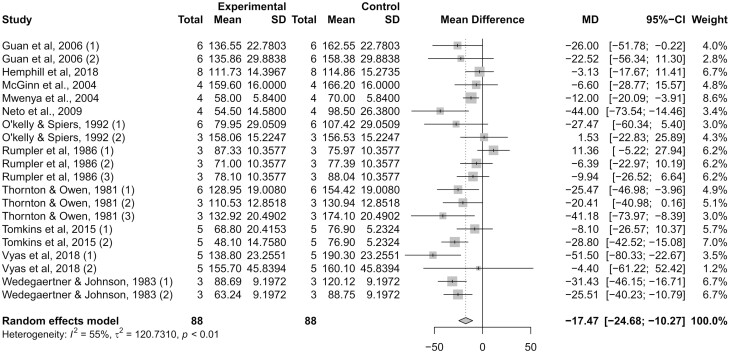
Forest plot reporting methane (CH4) production (g/d) in monensin-supplemented (experimental) and control (non-supplemented) groups. The number of experimental units, mean CH4 production, and SD are described for each treatment within studies, as well as the weight for each study to the meta-analysis. The dotted vertical line represents a standardized mean difference. Heterogeneity was calculated using *I²* and τ² statistics, and reported with the resultant *P*-value. Dietary inclusion of monensin decreased (*P *< 0.01) CH4 production by 17.47 g/d.

The studies included in this meta-analysis are described in Table 1, including sample size, dose of monensin, days receiving treatments prior to CH4 measurement, and dietary characteristics. The Egger’s regression test for funnel plot asymmetry was not significant (*P *= 0.26), indicating no publication bias as illustrated in Figure 1. When all studies are analyzed together (Figure 2), dietary inclusion of monensin decreased (*P *< 0.01) CH4 production by 17.5 g/d. This outcome represents a 15% reduction in CH4 production, supporting the values reported by Appuhamy et al. (2013). Despite the confidence interval ranging from 24.7 to 10.3 g/d in CH4 reduction by feeding monensin, moderate (*I²* = 55%; τ² = 120.7) heterogeneity was detected (*P *< 0.01) for CH4 production estimates between studies (Rabiee et al., 2010). Accordingly, additional meta-analyses were performed according to classes and our research hypothesis.

### Dose of monensin

**Figure 3.**
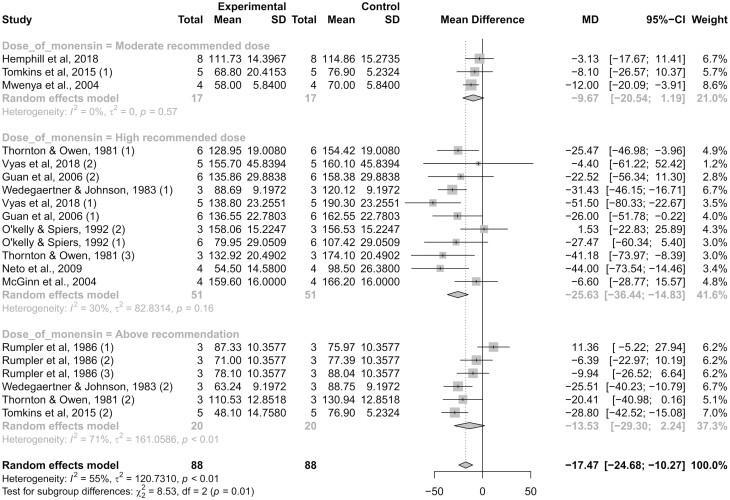
Forest plot reporting methane (CH4) production (g/d) in monensin-supplemented (experimental) and control (non-supplemented) groups. Studies were classified according to dose of monensin inclusion (dry matter [DM] basis; Duffield et al., 2012) as moderate recommended dose (less than 31 mg/kg of DM), high recommended dose (32 to 44 mg/kg of DM), and above recommendation (above 44 mg/kg of DM). The number of experimental units, mean CH4 production, and SD are described for each treatment within studies, as well as the weight for each study to the meta-analysis. The dotted vertical line represents a standardized mean difference. Heterogeneity was calculated using *I²* and τ² statistics, and reported within each class. Heterogeneity of studies across classes and the test for a class effect (subgroup differences) are also reported with the resultant *P*-value. Dietary inclusion of monensin decreased (*P *< 0.01) CH4 production in the high dose class, and tended to decrease (*P *≤ 0.07) CH4 production in the moderate and above recommendation classes.

Individual studies were classified based on dietary dose of monensin (MOD, HI, or ABV), and the reduction in CH4 production differed (*P *< 0.01) according to dosage class (Figure 3). The heterogeneity between studies within the MOD and HI classes were null (*I²* = 0%; τ² = 0; *P *= 0.57) or low (*I²* = 30%; τ² = 82.8; *P *= 0.16), respectively. Dietary inclusion of monensin at MOD averaged 17.7 mg/kg of diet DM, and tended (*P = *0.06) to decrease CH4 production by 9.7 g/d. Monensin inclusion at HI averaged 34.9 mg/kg of diet DM, and decreased (*P *< 0.01) CH4 production by 25.6 g/d. The heterogeneity between studies within the ABV class was substantial (*I*^*2*^ = 71%; τ² = 161.0; *P *< 0.01), which can be associated with the variation in MD between the Rumpler et al. (1986) studies caused by other factors besides monensin dose. Nonetheless, dietary monensin inclusion at this level averaged 54.4 mg/kg of diet DM, and tended to decrease (*P *= 0.07) CH4 production by 13.5 g/d.

Monensin limits availability of hydrogen for CH4 formation by increasing propionate to acetate ratio and reducing numbers of protozoa that generate hydrogen (Bergman et al., 1990; Ellis et al., 2012). Appuhamy et al. (2013) reported that monensin inclusion at ~32 mg/kg of diet DM yielded a greater reduction in CH4 production compared with ~21 mg/kg of diet DM, although this comparison was confounded by cattle type (dairy or beef, respectively). Others have described that monensin effects on CH4 production in beef and dairy are dose-dependent (Beauchemin et al., 2008; Ellis et al., 2012), with lower doses (i.e., less than 20 mg/kg of diet DM) having no effect in dairy cows and higher doses (24 to 35 mg/kg DM) reducing CH4 production in beef and dairy cattle. Results from this study corroborate that monensin decreases CH4 production in a dose-dependent manner, as the most effective response was noted when the HI dose was used. Nonetheless, monensin inclusion below 20 mg/kg of diet DM (MOD dose) also yielded anti-methanogenic effects, despite at a lesser extent (~62% less) compared with the HI dose. The reduction in anti-methanogenic response when monensin was added as ABV (~47% less) compared with HI was unexpected and warrants further investigation, as it was mostly caused by the studies of Rumpler et al. (1986).

### Length of monensin supplementation

**Figure 4.**
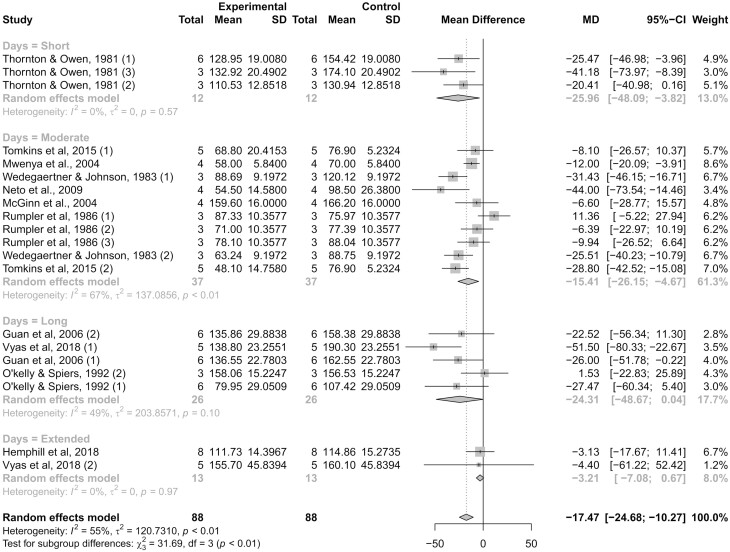
Forest plot reporting methane (CH4) production (g/d) in monensin-supplemented (experimental) and control (non-supplemented) groups. Studies were classified according to days receiving monensin until CH4 evaluation as short length (15 d), moderate length (23 to 33 d), long length (52 to 79 d), and extended length (94 and 161 d) based on ruminal dynamics in adapting to dietary changes (Brown et al., 2006). The number of experimental units, mean CH4 production, and SD are described for each treatment within studies, as well as the weight for each study to the meta-analysis. The dotted vertical line represents a standardized mean difference. Heterogeneity was calculated using *I²* and τ² statistics, and reported within each class. Heterogeneity of studies across classes and the test for a class effect (subgroup differences) are also reported with the resultant *P*-value. Dietary inclusion of monensin decreased (*P *≤ 0.05) CH4 production in the short, moderate, and long-length classes, and tended to decrease (*P *= 0.06) CH4 production in the extended-length class.

The interval between the beginning of monensin supplementation to the last CH4 measurement was used to classify studies (short, moderate, long, and extended lengths), and evaluate persistency of CH4 mitigation (Appuhamy et al., 2013). This latter trait is critical for development of dietary strategies aiming to mitigate CH4 emissions in beef cattle and other ruminant livestock species (Van Zijderveld et al., 2011). The reduction in CH4 production differed (*P *< 0.01) according to length class (Figure 4). The heterogeneity between studies was null for short (*I²* = 0%; τ² = 0; *P *≥ 0.57) and extended length classes, and low (*I²* = 49%; τ² = 203.8; *P *= 0.10) for the long length class. Monensin was supplemented for 15 d in Thornton and Owen (1981), which represented all three studies in the short-length class, and decreased (*P *= 0.03) CH4 production by 26.0 g/d. Within the long-length class, monensin was supplemented for an average of 64.6 d and decreased (*P *= 0.05) CH4 production by 24.3 g/d. Monensin was supplemented for an average of 127.5 d in the extended length class, and CH4 production tended (*P *= 0.06) to decrease by 3.21 g/d. The heterogeneity between studies classified as moderate length was moderate (*I²* = 67%; τ² = 137.1; *P *< 0.01), and caused by other factors that influenced CH4 reduction besides supplementation length. Nonetheless, monensin supplementation for an average of 26.5 d (moderate length) decreased (*P *= 0.01) CH4 production by 15.4 g/d.

Appuhamy et al. (2013) did not report an effect of monensin feeding duration on its anti-methanogenic effects in beef and dairy cows, despite inconsistent reports that such effects were either short-lived (Sauer et al., 1998; Guan et al., 2006) or long-lasting (O´Kelly & Spiers., 1992; Odongo et al., 2007). However, the meta-analysis by Appuhamy et al. (2013) did not include Vyas et al. (2018; study 2) and Hemphill et al. (2018), which supplemented monensin to beef cattle for 96 and 161 d, respectively. The anti-methanogenic effects of monensin in these studies were decreased by ≥80% compared with other length classes, suggesting that monensin is most effective in reducing CH4 production within the first 80 d of supplementation. Additional research is warranted to validate this latter rationale and characterize the persistency of CH4 mitigation when monensin is supplemented for extended periods (i.e., ≥ 90 d), which is relevant to commercial cow-calf, stocker/backgrounding, and feedlot operations.

### Diet Profile

**Figure 5.**
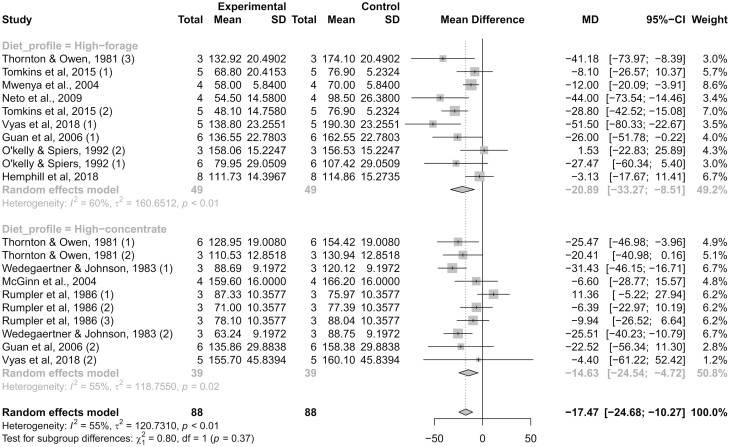
Forest plot showing reporting methane (CH4) production (g/d) in monensin-supplemented (experimental) and control (non-supplemented) groups. Studies were classified according to diet profile as high-forage (<50% concentrate inclusion, dry matter basis) or high-concentrate diets (≥50% concentrate inclusion, dry matter basis). The number of experimental units, mean CH4 production, and SD are described for each treatment within studies, as well as the weight for each study to the meta-analysis. The dotted vertical line represents a standardized mean difference. Heterogeneity was calculated using *I²* and τ² statistics, and reported within each class. Heterogeneity across classes and the test for a class effect (subgroup differences) are also reported with the resultant *P*-value. Dietary inclusion of monensin decreased (*P *< 0.01) CH4 production in both high-forage and high-concentrate diets.

Studies were classified according to dietary forage:concentrate ratio because high-forage diets often yield greater CH4 production in cattle (Beauchemin et al., 2008), and the anti-methanogenic effects of monensin were increased according to dietary fiber content (Appuhamy et al., 2013). However, the reduction in CH4 production did not differ (*P *= 0.37) when monensin was added to high-forage or high-concentrate diets (Figure 5), and the heterogeneity between studies within both classes was moderate (*I²* = 60 and 55%; τ² = 160.6 and 118.7; respectively; *P *≤ 0.02). The high-forage and high-concentrate diets averaged, respectively, 88:12 and 23:77 forage to concentrate ratio. Monensin inclusion decreased CH4 production by 20.89 g/d (*P *< 0.01) in cattle receiving the high-forage diets, and by 14.6 g/d (*P *< 0.01) in cattle receiving the high-concentrate diets. Grain fermentation in the rumen favors propiogenesis, which creates a natural hydrogen sink that may limit the anti-methanogenic activity of monensin (Murphy et al. 1982). Mitigation of CH4 was indeed decreased by 30% when monensin was included in high-grain diets, despite the nonsignificant class effect. These results corroborate the effectiveness of monensin in reducing CH4 production in beef cattle independent of diet profile, although such effects can be more pronounced when high-forage diets are used (Beauchemin et al., 2008; Appuhamy et al., 2013).

### Feeding Management

**Figure 6.**
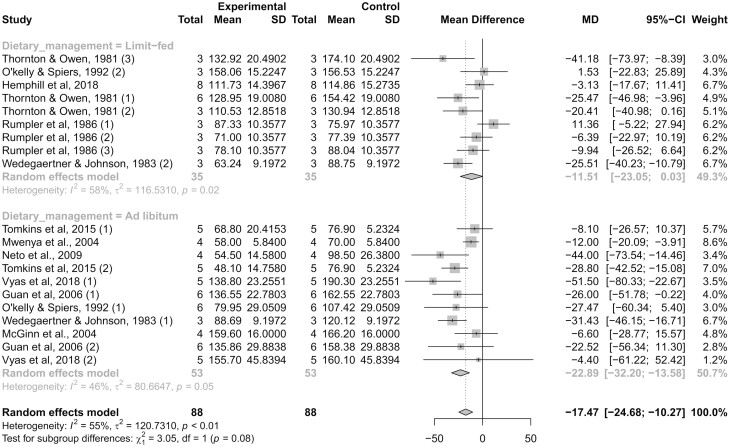
Forest plot showing reporting methane (CH4) production (g/d) in monensin-supplemented (experimental) and control (non-supplemented) groups. Studies were classified according to feeding management (ad libitum vs. limited-fed diets). The number of experimental units, mean CH4 production, and SD are described for each treatment within studies, as well as the weight for each study to the meta-analysis. The dotted vertical line represents a standardized mean difference. Heterogeneity was calculated using *I²* and τ² statistics, and reported within each class. Heterogeneity across classes and the test for a class effect (subgroup differences) are also reported with the resultant *P*-value. Dietary inclusion of monensin decreased (*P *≤ 0.05) CH4 production in both feeding managements (ad libitum vs. limited-fed diets).

Dry matter intake is also associated positively with CH4 production in cattle (Beauchemin et al., 2008), and appears to modulate the anti-methanogenic effects of monensin (Appuhamy et al., 2013). Studies included in this meta-analysis were classified according to feeding management (ad libitum vs. limited-fed diets), and the effects of monensin on CH4 production tended (*P *= 0.08) to differ between classes (Figure 6). The heterogeneity in the limited-fed class was moderate (*I²* = 58%; τ² = 116.5; *P *= 0.02), and low for the ad libitum class (*I²* = 46%; τ² = 80.6; *P *= 0.05). The inclusion of monensin decreased CH4 production by 22.9 g/d (*P *< 0.01) in cattle receiving diets for ad libitum intake, and by 11.5 g/d (*P *= 0.05) in cattle receiving limit-fed diets.

Appuhamy et al. (2013) reported that DM intake level did not affect the anti-methanogenic effects of monensin in beef cattle, and was negatively associated with the efficacy of monensin in mitigating CH4 in dairy cows. Results from this study suggest otherwise, as CH4 reduction doubled when monensin was added to diets fed ad libitum. The DM intake in studies feeding ad libitum diets averaged 2.04% of body weight (kg/kg basis), and 1.33% of body weight in the limit-fed studies. The difference in CH4 reduction between classes can be attributed to decreased availability of ruminal CH4 precursors when diets are limit-fed (Ellis et al., 2008), which restricts the anti-methanogenic potential and activity of monensin (Beauchemin et al., 2008). Hence, the effectiveness of monensin in mitigating CH4 production was impacted by feed offer regimen, which is relevant as both nutritional managements are used by research studies and commercial beef production systems (Galyean and Hales, 2023).

## Conclusions

This meta-analysis provides novel insights into the anti-methanogenic effects of monensin on beef cattle. Monensin inclusion decreased in vivo CH4 production by 17.5 g/d across all studies, representing a 15% reduction in CH4 emission. The anti-methanogenic effectiveness of monensin, however, was modulated by dosage, length of supplementation, and diet characteristics. The most effective responses of monensin inclusion were observed: (1) during the first 79 d of monensin supplementation, (2) when monensin was included between 32 to 44 mg/kg of diet DM, (3) when monensin was added to high-forage diets, and (4) when monensin was added to diets offered for ad libitum intake. Additional research is warranted to characterize persistency of CH4 mitigation beyond ~90 d of monensin supplementation. Collectively, this study corroborates monensin as nutritional strategy to mitigate CH4 production in beef cattle operations.
